# Characterization of Autozygosity in Pigs in Three-Way Crossbreeding

**DOI:** 10.3389/fgene.2020.584556

**Published:** 2021-01-28

**Authors:** Audrey Ganteil, Silvia T. Rodriguez-Ramilo, Bruno Ligonesche, Catherine Larzul

**Affiliations:** ^1^GenPhySE, Université de Toulouse, INRAE, ENVT, Castanet-Tolosan, France; ^2^SAS NUCLEUS, Le Rheu, France

**Keywords:** runs of homozygosity, genomic inbreeding, crossbreeding, swine, genomic diversity

## Abstract

Crossbreeding in livestock can be used to increase genetic diversity. The resulting increase in variability is related to the heterozygosity of the crossbred animal. The evolution of diversity during crossbreeding can be assessed using genomic data. The objective of this study was to describe patterns of runs of homozygosity (ROH) in animals resulting from three-way crossbreeding, from parental pure lines, and in their crossbred offspring. The crossbreeding scheme consisted of a first crossbreeding Pietrain boars and Large White sows, after which the offspring of the Pietrain × Large White were crossed with Duroc boars. The offspring of the second crossbreeding are called G0, the offspring of G0 boars and G0 sows are called G1. All the animals were genotyped using the Illumina SNP60 porcine chip. After filtering, analyses were performed with 2,336 animals and 48,579 autosomal single nucleotide polymorphism (SNP). The mean ROH-based inbreeding coefficients were shown to be 0.27 ± 0.05, 0.23 ± 0.04, and 0.26 ± 0.04 for Duroc, Large White, and Pietrain, respectively. ROH were detected in the Pietrain × Large White crossbred but the homozygous segments were fewer and smaller than in their parents. Similar results were obtained in the G0 crossbred. However, in the G1 crossbreds the number and the size of ROH were higher than in G0 parents. Similar ROH hotspots were detected on SSC1, SSC4, SSC7, SSC9, SSC13, SSC14, and SSC15 in both G0 and G1 animals. Long ROH (>16 Mb) were observed in G1 animals, suggesting regions with low recombination rates. The conservation of these homozygous segments in the three crossbred populations means that some haplotypes were shared between parental breeds. Gene annotation in ROH hotspots in G0 animals identified genes related to production traits including carcass composition and reproduction. These findings advance our understanding of how to manage genetic diversity in crossbred populations.

## 1. Introduction

Crossbreeding exploits genetic diversity between breeds with different objectives including the contribution of new genes, the heterosis effect, complementarity between production traits, and increased genetic variability (Bidanel, 1992). Increase in genetic variability in crossbred animals is related to their heterozygous status. Crossbred animals become heterozygous for all loci when parental breeds are homozygous for a different allele. When crossbreeding is used to create a new synthetic line, two or more parental breeds can be crossed. Crossbred offspring can be mated among themselves at each generation. After several generations, the animals will become genetically homogeneous and this population can be considered a new line. One important point is the management of genetic diversity during this process. In this context, characterizing genetic diversity with pedigree data is impossible because genealogical relationships among parental breeds used in the crossbreeding cannot be established. However, genomic data can be analyzed to overcome the problem (Zhang et al., 2019).

Genomic-based inbreeding coefficients can be computed to provide information about diversity in a population. In a recent study, Schäler et al. (2020) distinguished between four different approaches to calculate the coefficients: variance of additive genetic values, single nucleotide polymorphism (SNP) homozygosity, uniting gametes, and runs of homozygosity (ROH). The first three coefficients depend on estimating allele frequencies in the population, contrary to ROH-based inbreeding coefficients. ROH-based inbreeding coefficients are of real interest in crossbred populations with high levels of heterozygosity, because inbreeding coefficients calculated using intermediate allele frequencies are close or equal to 0 (Zhang et al., 2015).

In a diploid genome, ROH are continuous stretches of homozygous genotypes, and their quantification reflects autozygosity (Ferenčaković et al., 2013; Peripolli et al., 2017). Autozygosity occurs when the two parents of an individual have at least one common ancestor. ROH can be influenced by genetic drift, genetic bottlenecks, mating between relatives, or intensive selection (Peripolli et al., 2017). ROH are not distributed evenly along the genome. Pemberton et al. (2012) defined two types of regions in terms of ROH distribution: hotspots, with a high frequency of ROH, and coldspots, with a low frequency. Hotspots show a loss of diversity compared to coldspots. In pig, Bosse et al. (2012) showed that ROH distribution can be influenced by demographic phenomena and the chromosomal recombination landscape. An ROH gene content analysis in the same study showed that only a few ROH are under positive selection.

The study of ROH in crossbred animals provides information on the genomic similarities between parental lines. ROH shared between two porcine breeds has already been demonstrated in Large White and Landrace pigs (Zanella et al., 2016). Persistence of ROH in crossbred pigs has been reported in real animals in two-way crossbreeding (Landrace × Large White) and in simulated animals in three-way crossbreeding [Duroc × (Landrace × Large White)] (Howard et al., 2016; Gómez Raya et al., 2019). These results indicate that similar haplotypes were selected in porcine breeds and can persist in crossbred offspring.

The objective of this study was to analyze ROH patterns during three-way crossbreeding aimed at creating a new porcine line. ROH were searched for individuals resulting from three parental pure breeds and their offspring over two generations in order to characterize and compare autozygosity among pure breeds, and to monitor the modification of ROH in the crossbreed.

## 2. Materials and Methods

### 2.1. Genotyped Animals

**Figure 1:**
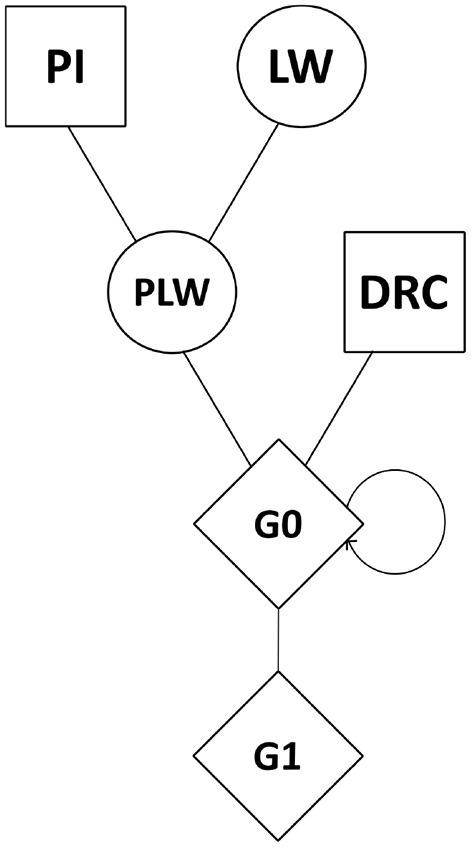
Crossbreeding scheme. Squares represent males, circles represent females, and diamonds represent unspecified gender. DRC, Duroc; G0, (Pietrain × Large White) × Duroc offspring; G1, G0 × G0 offspring; LW, Large White; PI, Pietrain; PLW, Pietrain × Large White.

Genomic data were obtained from the breeding company NUCLEUS (Le Rheu, France) from a three-way crossbreeding protocol (Figure 1). Animals from three pure lines were genotyped: 80 Pietrain (PI) boars, 240 Large White (LW) sows, and 89 Duroc (DRC) boars. Crossbred animals were also genotyped: Pietrain × Large White crossbred offspring (442 PLW sows), Duroc × PLW crossbred offspring (69 G0 boars and 471 G0 sows) and G0 × G0 crossbred offspring (472 G1 boars and 473 G1 sows). Genotyping was carried out by the Labogena laboratory using the Illumina Porcine Chip, Porc_XT_60K. We used a reference map based on the *Sus scrofa* 11.1 pig genome assembly. Quality control of genotypes was performed with PLINK v1.9 software (Chang et al., 2015). Only markers on autosomes were kept. Markers with more than 5% of missing genotypes were discarded. We checked that all the animals had more than 90% genotyped markers. No minor allele frequency (MAF) pruning was used here according to Meyermans et al. (2020). After quality control, 2,336 animals and 48,579 SNP were retained for analysis.

### 2.2. Population Structure Analysis

First, a multidimensional scaling analysis (MDS) was conducted to visualize the genetic distances between animals and the structure of the pig population using PLINK v1.9 software. After this we computed Cockerham and Weir (1984) *F*_*ST*_ analysis with PLINK v1.9 software to quantify genetic differentiation among pig groups. Finally, an admixture analysis was performed with ADMIXTURE v1.3.0 software (Alexander et al., 2009). Here, the number of genetic populations considered was 3 (for *K* parameter), the number of pure breeds involved in the crossbreeding.

### 2.3. Detection of Runs of Homozygosity

ROH were detected with PLINK v1.9 software. First, to choose the minimum size to define an ROH (in terms of SNP and kb) and the minimum SNP density in an ROH, we selected a range of minimum numbers of SNP and minimum size in kb according to Peripolli et al. (2017). Tests of parental populations (Pietrain and Large White) were then performed to choose the values that neither underestimated nor overestimated the number of ROH detected (Ganteil et al., 2020). The values selected to define an ROH were 30 SNP and 1,000 kb and the minimum density was set at one SNP per 100 kb. Regarding the parameters for the number of SNP in the sliding window, Curik et al. (2014) recommended using a sliding window equal or larger than the minimum size used to define an ROH. We thus decided to set the sliding window at 30 SNP. We allowed one missing SNP per sliding window. To obtain strictly homozygous ROH, no heterozygous SNP were allowed per sliding window. All the other parameters available in PLINK that are not mentioned above were default settings.

The ROH were also divided into three classes based on length: 1–8, 8–16, and >16 Mb corresponding to small, medium, and large ROH, respectively.

### 2.4. Estimation of ROH-Based Inbreeding

Genomic analyses after detection of ROH were performed with the R package DetectRUNS (Biscarini et al., 2019). We calculated the ROH-based inbreeding coefficient (*F*_*ROH*_) for each animal as:

(1) $$\begin{array}{l} F_{ROH}=\frac{\sum L_{ROH}}{L_{autosomes}} \end{array}$$

where ∑*L*_*ROH*_ is the sum of the length of all the ROH detected in an animal in bp, and *L*_*autosomes*_ is the total length of the autosomes covered by markers in bp.

The most frequent SNP in ROH are ROH hotspots. To define the ROH hotspots, we first computed the frequency at which each SNP is detected in an ROH in each pure breed and crossbred population. Then, using the method proposed by Purfield et al. (2017), we selected the top 1% of SNP observed in an ROH in each pure breed and crossbred population and adjacent SNP above this threshold were merged into genomic regions corresponding to ROH hotspots.

### 2.5. Genomic Annotation

Genomic annotation was performed in G0 crossbreds, the first generation of the new line. In this generation, ROH hotspots mean frequent haplotype sharing between Pietrain, Large White, and Duroc. Genes in ROH hotspots in G0 animals were extracted using Biomart on the Ensembl website (https://www.ensembl.org/biomart/martview/fbef5263e7166fc734235c9325399e4d, version 100 released in April 2020). As dataset, we used the current pig genome assembly (build 11.1), and the regions of interest on the chromosomes were used as a filter to extract gene symbols.

## 3. Results

### 3.1. Population Genetic Structure

**Figure 2:**
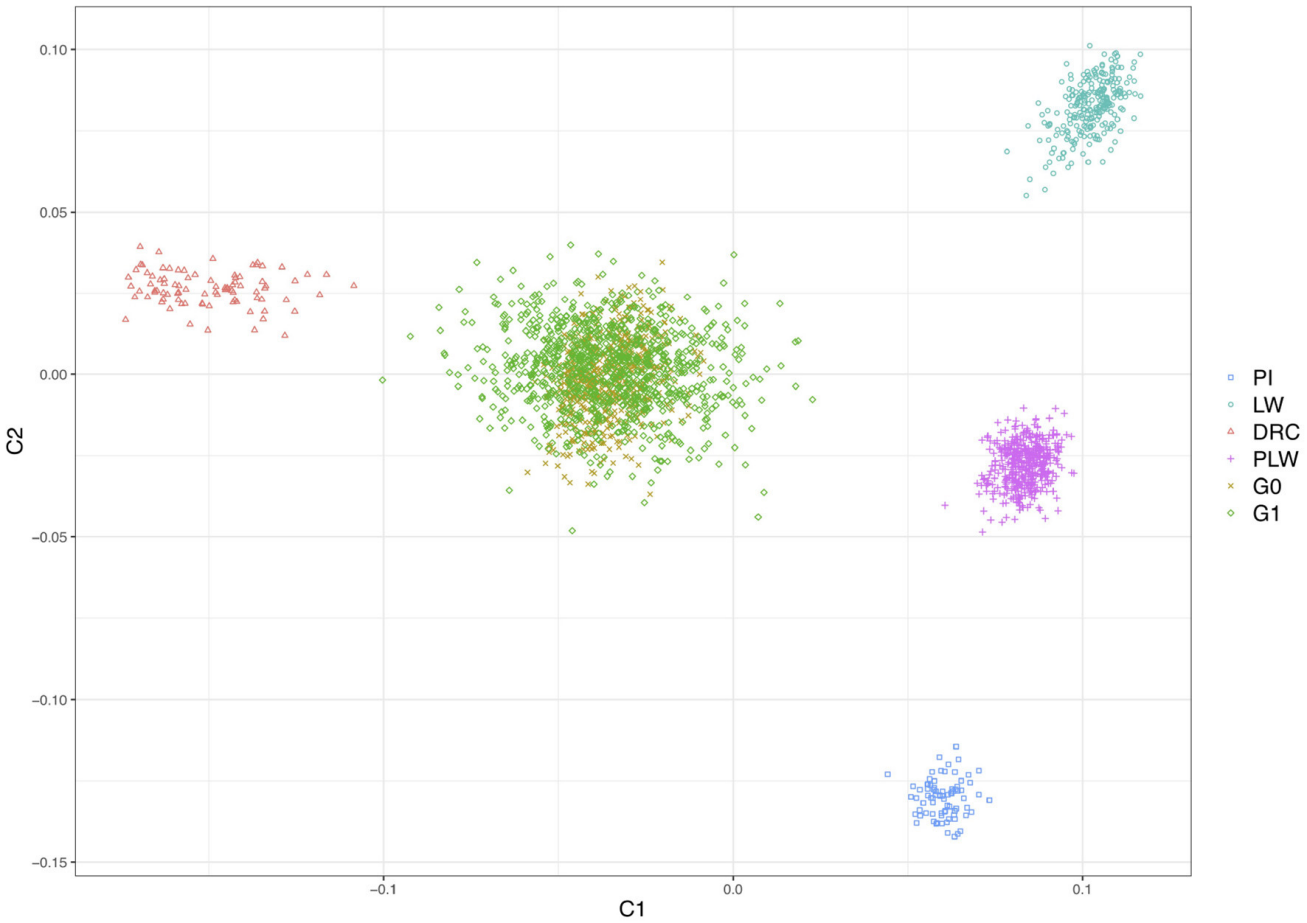
Population structure shown in a multidimensional scaling analysis (MDS) plot of all animals. DRC, Duroc; G0, (Pietrain × Large White) × Duroc offspring; G1, G0 × G0 offspring; LW, Large White; PI, Pietrain; PLW, Pietrain × Large White.

Figure 2 shows the genetic distances between each animal. The three founder populations, Pietrain, Large White and Duroc, were well-separated and distant populations. The crossbred PLW are halfway between Pietrain and Large White populations. This result is consistent with the chromosome composition of PLW: half Pietrain and half Large White. The first axis separates the Pietrain, Large White, and PLW populations from Duroc. The G0 and G1 crossbred are plotted in the center of the MDS plot halfway between Duroc and PLW. G0 animals were more grouped than G1 animals, which were more spread out in the center of the MDS plot. This result highlights random segregation and recombination of chromosomes during meiosis. Thus, G1 animals all inherited in different proportions of Duroc, Pietrain, and Large White chromosomal segments. In addition, new original combinations of alleles from the 3 parental breeds are present in this generation. These results illustrate a generation of genetic diversity between G0 and G1 animals.

**Figure 3:**
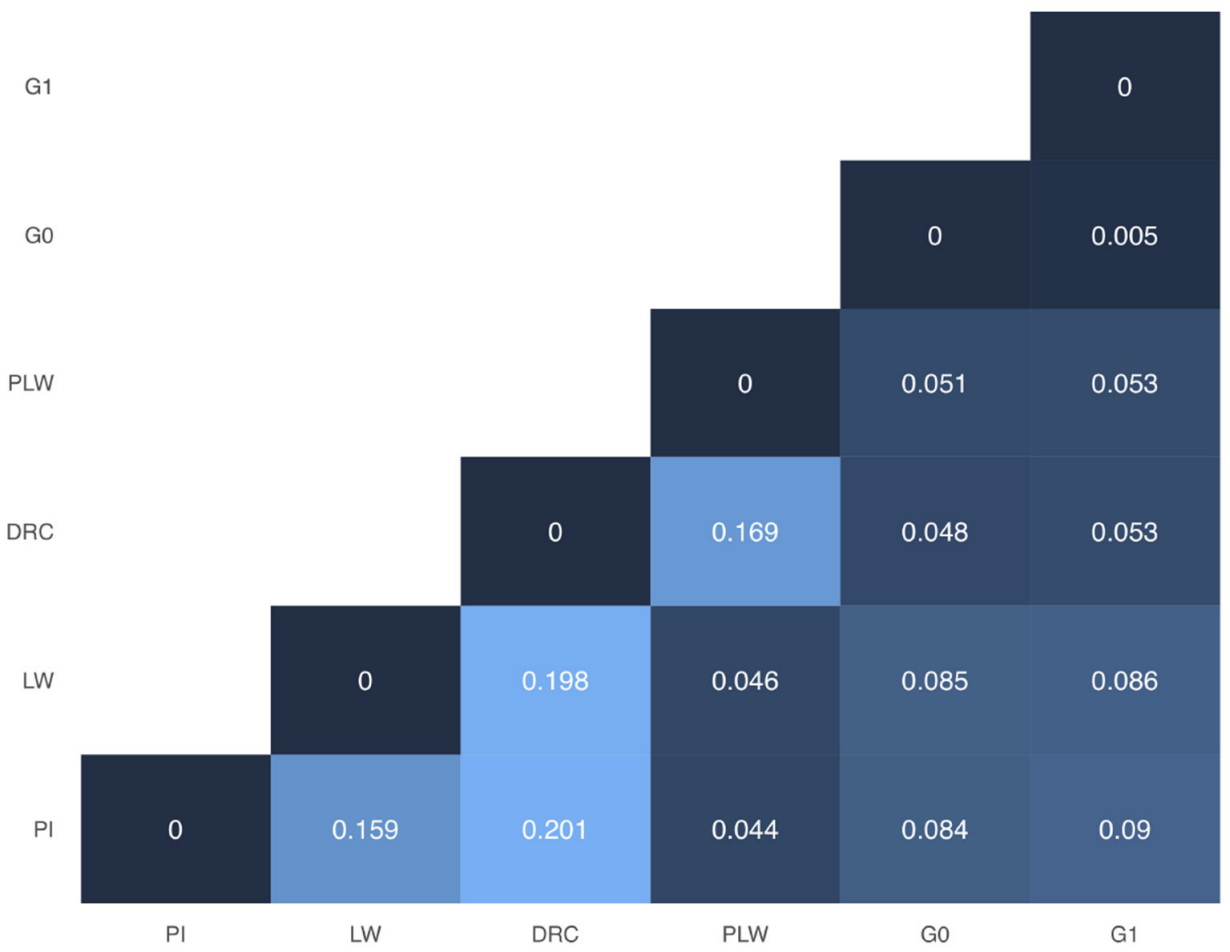
Weir and Cockerham *F*_*ST*_ heatmap for all groups. DRC, Duroc; G0, (Pietrain × Large White) × Duroc offspring; G1, G0 × G0 offspring; LW, Large White; PI, Pietrain; PLW, Pietrain × Large White.

In Figure 3, we presented the pairwise Weir and Cockerham' *F*_*ST*_ values between all purebred and crossbred populations. Among the pure breeds, we observed the highest differentiation coefficients between Duroc and Pietrain and Duroc and Large White (0.201 and 0.198, respectively). Pietrain and Large White are less genetically differentiated with a *F*_*ST*_ value of 0.159. Between crossbred offspring and their parental pure breeds, we observed *F*_*ST*_ values ranged between 0.044 and 0.09. Concerning G0 and G1 crossbred, they have the lowest observed *F*_*ST*_ value.

**Figure 4:**
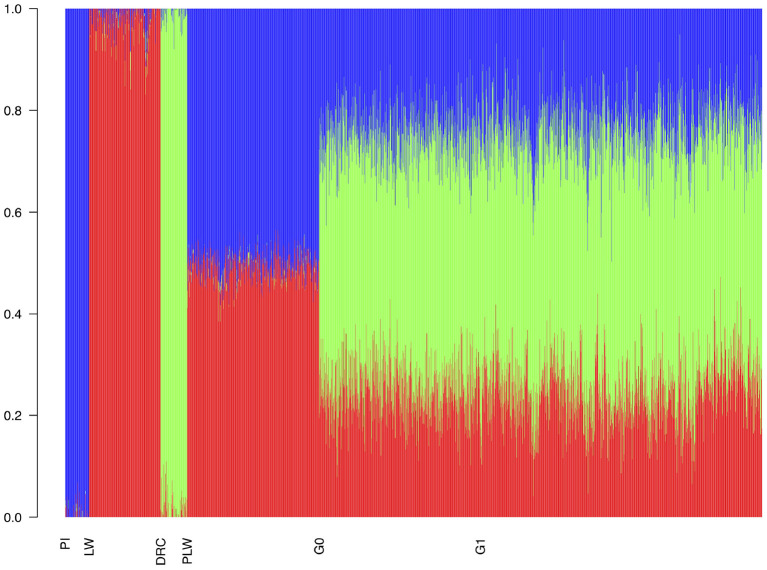
Admixture analysis of each population from the three-way crossbreeding. The number of clusters was set to *K* = 3. DRC, Duroc; G0, (Pietrain × Large White) × Duroc offspring; G1, G0 × G0 offspring; LW, Large White; PI, Pietrain; PLW, Pietrain × Large White.

With the admixture analysis, we can validate the crossbreeding scheme (Figure 4). We observed the admixture of the crossbred populations based on 3 different genetic origins. PLW animals were half Pietrain and half Large White. After, G0 and G1 animals presented similar profiles of admixture, approximately a quarter Pietrain, a quarter Large White, and a half Duroc.

### 3.2. ROH Patterns

**Figure 5:**
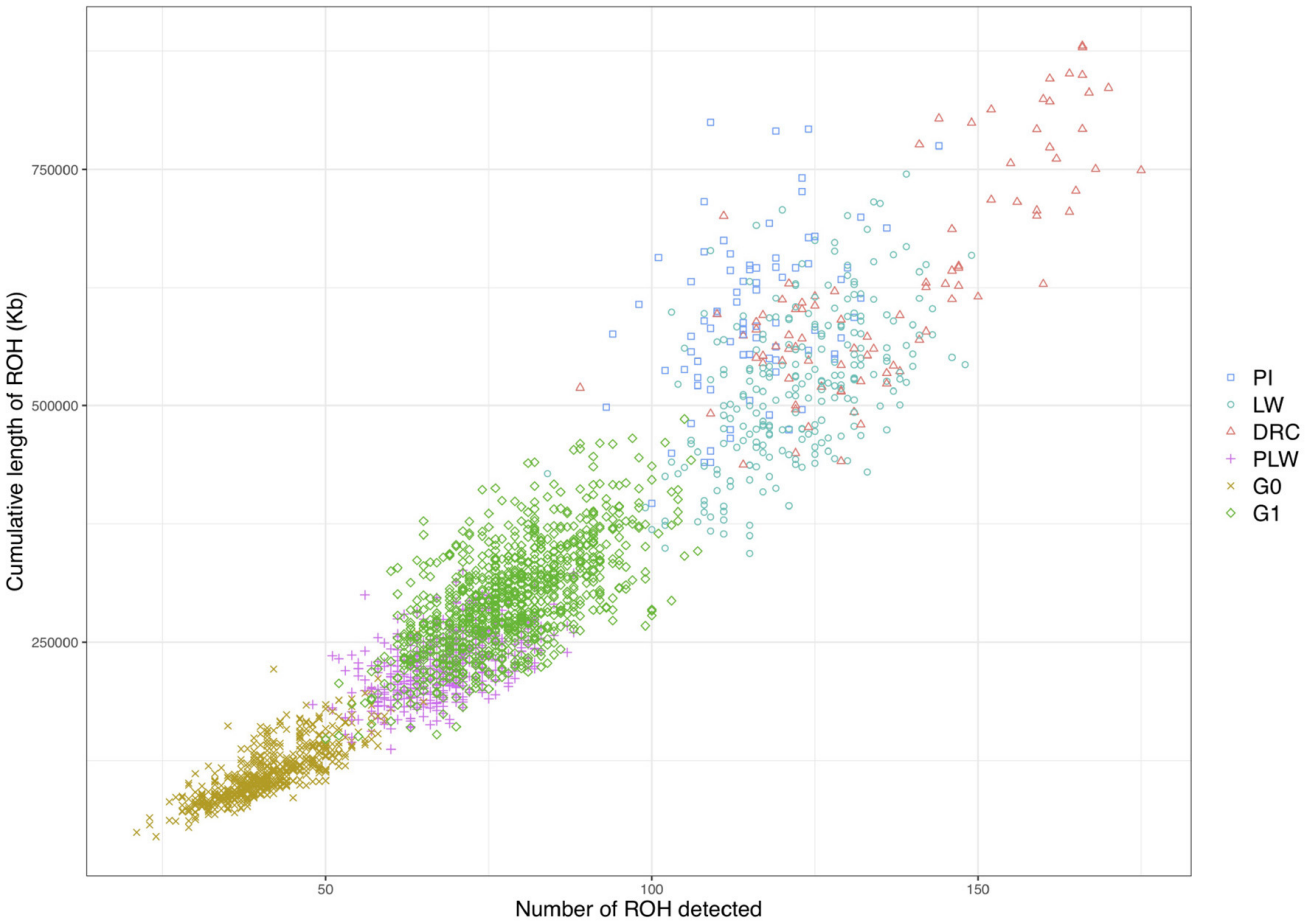
Individual pattern of runs of homozygosity (ROH). The cumulative length of ROH is plotted against the number of ROH detected for each animal. DRC, Duroc; G0, (Pietrain × Large White) × Duroc offspring; G1, G0 × G0 offspring; LW, Large White; PI, Pietrain; PLW, Pietrain × Large White.

We observed different ROH patterns among the 3 pure breeds and 3 crossbred populations studied (Figure 5). The three pure breeds had both the greater cumulative ROH length and more ROH than the crossbred animal. ROH persisted in the three crossbred populations due to haplotypes shared between parental breeds. The most ROH and the longest cumulative size were observed in Duroc animals. Pietrain and Large White animals had similar numbers of ROH, whereas Pietrain tended to have higher cumulative length, which means that these animals had larger ROH than Large White. G1 animals had the most ROH and the longest cumulative size of ROH of the three crossbred populations, and G0 animals had the smallest number of ROH and the lowest cumulative size. PLW animals were between the two.

**Figure 6:**
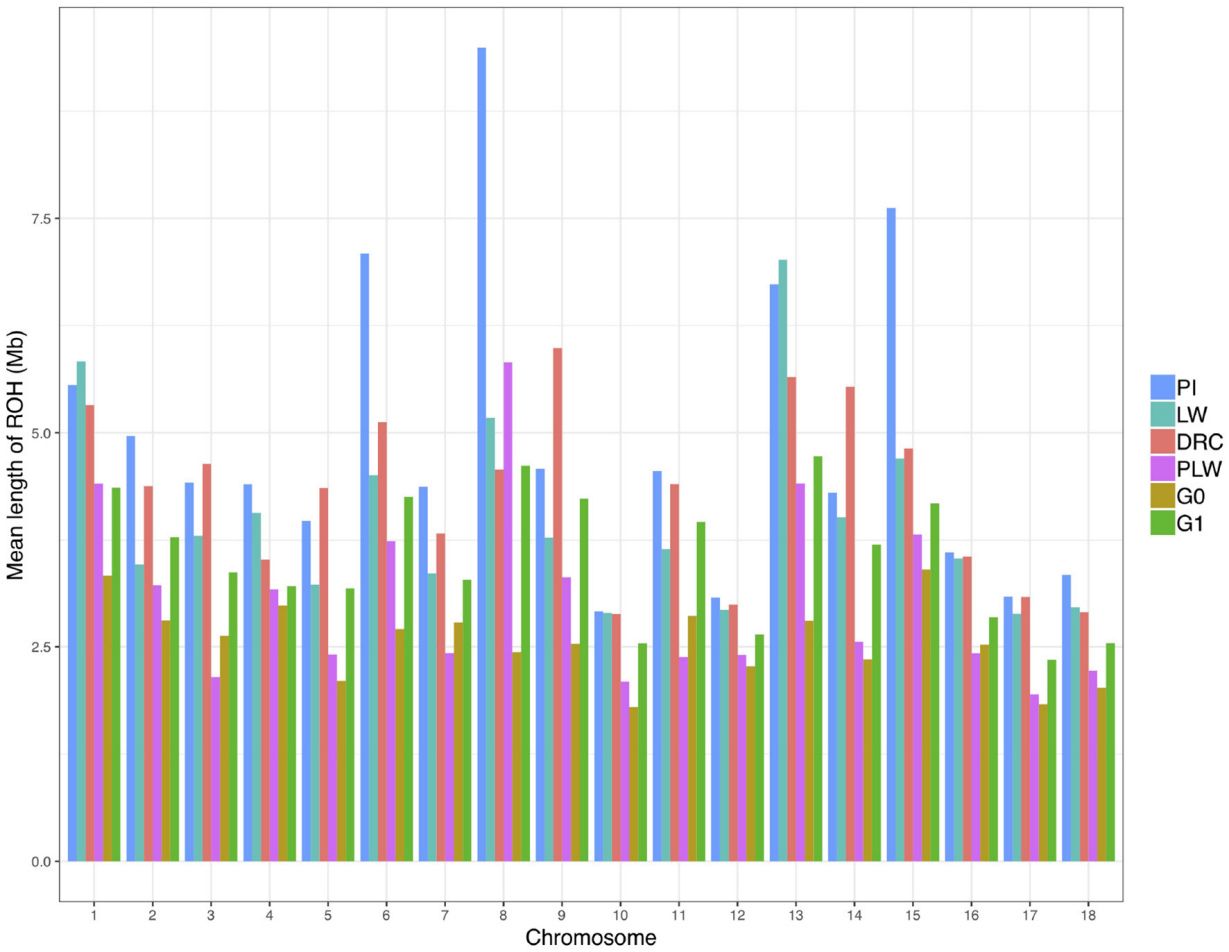
Mean length of runs of homozygosity (ROH) detected per chromosome and group. DRC, Duroc; G0, (Pietrain × Large White) × Duroc offspring; G1, G0 × G0 offspring; LW, Large White; PI, Pietrain; PLW, Pietrain × Large White.

We observed the mean length of ROH detected per chromosome for each pig population (Figure 6). Pure breeds presented the highest mean length of ROH along the chromosomes. Pietrain animals had the highest observed mean length of ROH in particular for SSC6, SSC8, and SSC15 compared to other groups. For crossbred animals, in all chromosomes, G1 had a mean length of ROH greater than G0.

**Figure 7:**
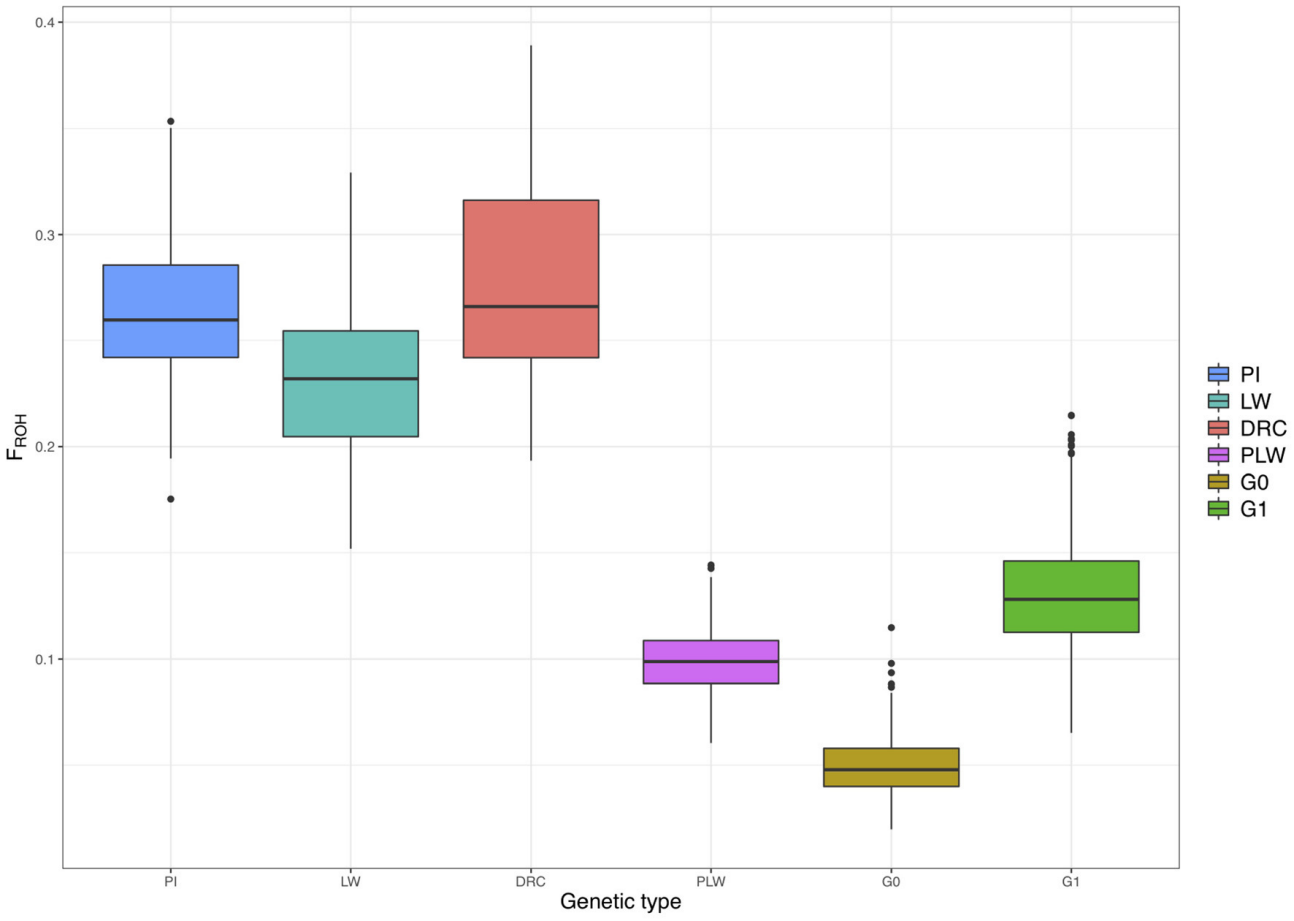
Runs of homozygosity (ROH)-based inbreeding coefficient (*F*_*ROH*_) for each genetic type. DRC, Duroc; G0, (Pietrain × Large White) × Duroc offspring; G1, G0 × G0 offspring; LW, Large White; PI, Pietrain; PLW, Pietrain × Large White.

Figure 7 shows the ROH-based inbreeding coefficient (*F*_*ROH*_) for each pure breed and crossbred population. As expected, average *F*_*ROH*_ was lower in the crossbred individuals (PLW, G0, and G1) than in the pure breeds (PI, LW, and DRC). The average *F*_*ROH*_ for each group was 0.27 ± 0.05, 0.26 ± 0.04, 0.23 ± 0.04, 0.13 ± 0.02, 0.10 ± 0.01, and 0.05 ± 0.01 for Duroc, Pietrain, Large White, G1, PLW, and G0, respectively.

### 3.3. ROH Hotspots

**Figure 8:**
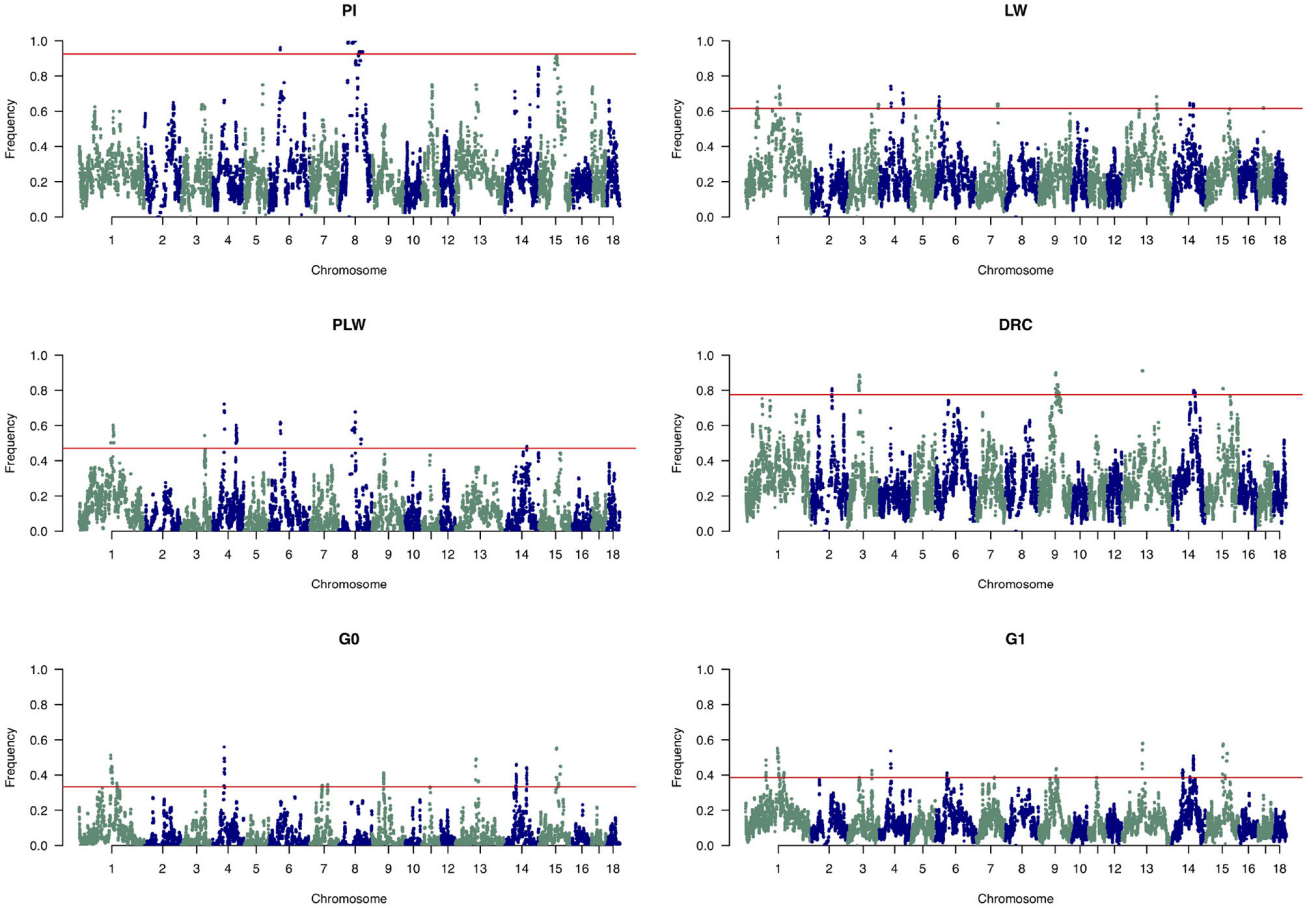
Manhattan plots of the frequency of SNP detected inside a runs of homozygosity (ROH). The horizontal line indicates the cutoff level for ROH hotspot detection in each genetic group. It corresponds to the top 1% SNP most frequently observed in an ROH in each pure breed and crossbred population. DRC, Duroc; G0, (Pietrain × Large White) × Duroc offspring; G1, G0 × G0 offspring; LW, Large White; PI, Pietrain; PLW, Pietrain × Large White.

Figure 8 shows the frequency of a single SNP detected inside an ROH along the genome. The exact genomic position of ROH hotspots are reported in Supplementary Table 1. The occurrences of ROH varied among the three pure breeds along the genome. ROH hotspots were identified in Duroc animals on SSC2, SSC3, SSC9, SSC13, SSC14, and SSC15, and in Pietrain animals on SSC6 and SSC8. Finally, in Large White animals, ROH hotspots were identified on SSC1, SSC3, SSC4, SSC6, SSC7, SSC13, SSC14, and SSC17. Some SNP were located in ROH particularly on SSC8, in all Pietrain animals. Among crossbred animals, PLW animals presented ROH hotspots on SSC1, SSC3, SSC4, SSC6, SSC8, and SSC14. G0 and G1 animals had ROH hotspots located close together, especially on SSC1, SSC4, SSC7, SSC9, SSC13, SSC14, and SSC15. These results highlight regions of the genome where there is high probability of haplotype sharing between the three parental breeds.

### 3.4. ROH Size Categories

**Figure 9:**
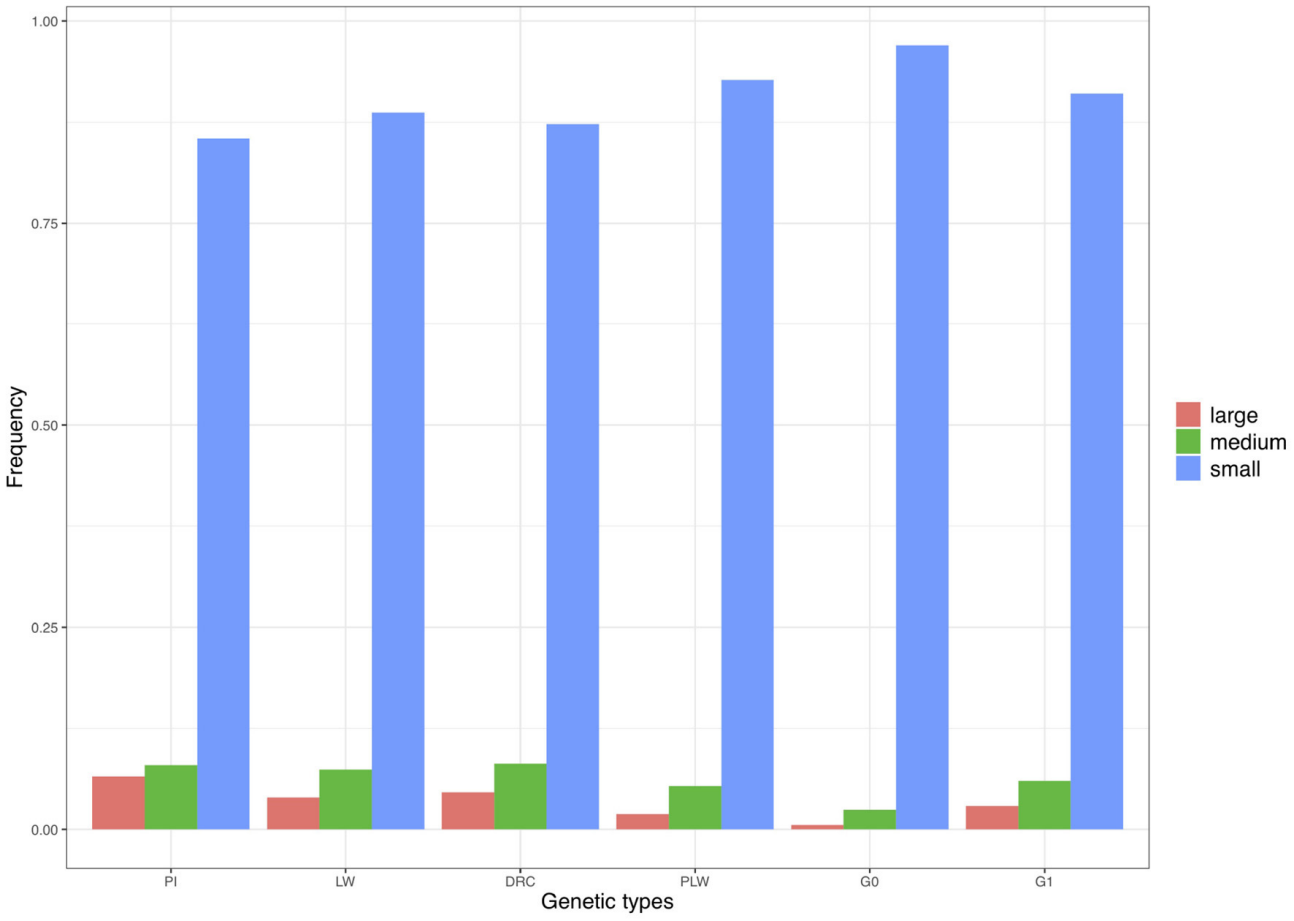
Frequency of runs of homozygosity (ROH) in the three size classes per genetic type. DRC, Duroc; G0, (Pietrain × Large White) × Duroc offspring; G1, G0 × G0 offspring; LW, Large White; PI, Pietrain; PLW, Pietrain × Large White.

We divided the homozygous segments into three size classes: small, medium, and large (Figure 9). The small category was the most widely represented across the pure breeds and crossbred populations. The highest frequency of small ROH was observed in the G0 population and the lowest in the Pietrain population. Minimum frequencies of the two other size classes were observed in G0. The three pure breeds showed the highest level of ROH in the medium and large classes. Among the three crossbred populations, G1 animals had the highest proportion of medium and large ROH.

**Figure 10:**
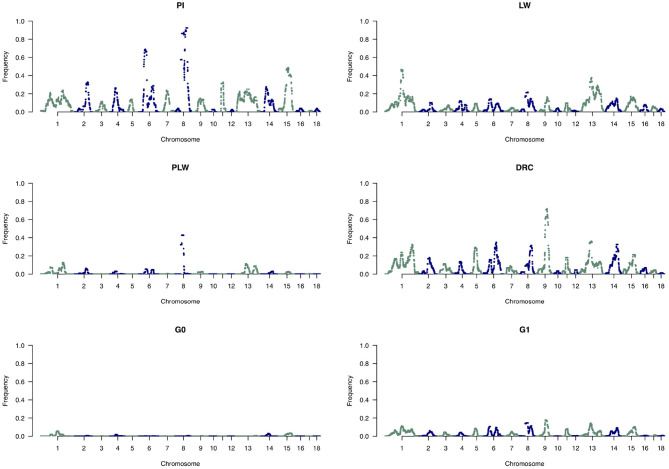
Manhattan plots of the frequency of SNP detected inside large runs of homozygosity (ROH). DRC, Duroc; G0, (Pietrain × Large White) × Duroc offspring; G1, G0 × G0 offspring; LW, Large White; PI, Pietrain; PLW, Pietrain × Large White.

To analyze the distribution of large ROH in more detail, we only used the frequency of SNP detected in large ROH (Figure 10). In the Pietrain breed, we detected two frequent chromosomal regions with large ROH on SSC6 and SSC8 shared between more than 60 and 80% of animals, respectively. In Large White, large ROH were located on SSC1 and SSC13, and in Duroc animals on SSC9. Like Pietrain, the PLW crossbred had long ROH located on SSC8. G0 had no chromosomal regions with frequent large ROH, but in their offspring (G1) we observed a slight increase in large ROH on many chromosomes, for example, SSC1, SSC6, SSC8, SSC9, SSC13, SSC14, and SSC15.

### 3.5. Relation Between ROH and Gene Annotation

Among the ROH hotspots in G0 animals (Figure 8), we first selected hotspots larger than 1 Mb. Thereafter, we kept only ROH hotspots with an average frequency of detection of SNP in ROH greater than 0.40. Seven ROH hotspots were kept on SSC1, SSC4, SSC13, SSC14, and SSC15. The size of the regions ranged from 1.45 Mb (SSC14) to 7.26 Mb (SSC1) (Table 1). We extracted the list of genes detected in the ROH hotspots and we reviewed the literature on these genes to find information that could be related to pig production. Thus, we identified 24 genes of interest in these hotspots. They were associated with production traits that could have been under similar selection in the three founder breeds.

**Table 1:** Runs of homozygosity (ROH) hotspots in G0 and putative genes of interest under similar selection in the three founder breeds.

**Chromosome**	**Positions (Mb)**	**Size (Mb)**	**Number of SNP**	**Genes of interest**
1	132.98–140.24	7.26	101	*IGF1R, MEF2A, ALDH1A3, LRRK1*
4	48.06–50.94	2.88	37	*MMP16, CNGB3, CPNE3, RMDN1, WWP1, SLC7A13, ATP6V0D2*
13	84.93–88.06	3.13	43	*PLOD2*
14	46.83–48.28	1.45	45	*LIF, GAL3ST1, INPP5J, PLA2G3*
14	89.96–91.65	1.69	49	*ALOX5*
15	72.38–75.16	2.78	39	*XIRP2, B3GALT1, STK39, CERS6*
15	88.36–91.57	3.21	34	*NCKAP1*

*ROH hotspots listed here are larger than 1 Mb with an average frequency of detection of SNP in ROH greater than 0.40*.

## 4. Discussion

To our knowledge, this is the first ROH characterization in a three-way crossbreeding program with the aim of creating a new synthetic pig line. The objective of a new line is to combine the qualities of several parental breeds in a new synthetic breed. In this context, managing diversity is a major constraint to long-term genetic progress. Studying ROH during the creation of a new line is a useful way to characterize the existing diversity in founder pure breeds and the resulting diversity in the crossbred animals in the new line.

### 4.1. Autozygosity in the Purebred

The three pure breeds had relatively similar *F*_*ROH*_. Other authors have already compared ROH patterns of different breeds. These studies are difficult to compare because population samples differ in origin and size, and the parameters used for the detection of ROH may greatly influence the results (Meyermans et al., 2020). However, we observed large ROH in pure lines, as already described in other studies (Bosse et al., 2015; Howard et al., 2016; Gorssen et al., 2019). Large ROH correspond to recent inbreeding (Curik et al., 2014), which is expected to be more harmful than ancient inbreeding, because selection has had time to reduce the frequency of deleterious alleles that are purged over time (Doekes et al., 2019).

ROH hotspots were not uniformly distributed along the genome across the three pure breeds. Consequently, ROH hotspots in the genome may highlight signatures of selection in pure breeds. Four ROH hotspots were detected in the central region of SSC8 in Pietrain. Moreover, this region contained large ROH (≥16 Mb) as already highlighted in other studies on Pietrain populations (Bosse et al., 2015; Gorssen et al., 2019). One of hypotheses proposed by these authors is the presence of a selection signature in this region. We showed that Large White shared similar haplotypes in SSC8 with Pietrain because we detected ROH in PLW animals. But this region seems less fixed in Large White than in Pietrain. Another hypothesis to explain this ROH pattern could be limited recombination in this region, which is close to the center of SSC8. In pig, this chromosome is metacentric (Raudsepp and Chowdhary, 2011). Previous studies showed that regions with high chromosomal recombination rates tend to be close to telomeres, and close correlations between ROH distribution or size with recombinations and GC content have already been observed in pig (Bosse et al., 2012; Tortereau et al., 2012). The regions with low recombination rates on SSC8 identified by Tortereau et al. (2012) include almost all the ROH hotspots detected in our Pietrain population. However, these low recombination rates did not generate ROH hotspots in Duroc and Large White. More information about the biological functions of the genes located in this region is needed to better understand this specific ROH pattern in Pietrain. However, ROH hotspots cover a large chromosomal region on SSC8 making gene detection more difficult to interpret. Studying the evolution of these hotspots with crossbreeding between Pietrain and other porcine breeds would be a good way to monitor the evolution of ROH in the second generation and to analyze recombination events. In fact, the persistence of large ROH segments in crossbred offspring suggests the absence of recombination in these ROH (Bosse et al., 2012).

### 4.2. Autozygosity in the Three Crossbred Populations

ROH were also detected in crossbred individuals. Our results confirm those of previous studies of the persistence of ROH in crossbred animals (Howard et al., 2016; Gómez Raya et al., 2019), where the existence of ROH is explained by haplotype sharing between parental breeds. PLW animals had a higher *F*_*ROH*_ than G0 animals. Moreover, the G0 population presented the lowest level of autozygosity among the crossbred. As expected, the maximum diversity during the constitution of this new line appeared to be achieved in this generation. In PLW, ROH are generated by haplotype sharing between Pietrain and Large White and in G0 by haplotype sharing between Pietrain and Duroc or Large White and Duroc. Gómez Raya et al. (2019) showed that the correlation between the probability of autozygosity and the genetic differentiation (*F*_*ST*_) of breeds was negative. Consequently, Pietrain and Large White may be genetically closer than Pietrain and Duroc or Large White and Duroc. To support this hypothesis, we analyzed *F*_*ST*_ in our three pure breeds. The differentiation between Duroc and Pietrain or Duroc and Large White was higher than that between Pietrain and Large White. These results are consistent with the *F*_*ST*_ obtained by Gorssen et al. (2019). Moreover, genetic distance between these three breeds has already been analyzed (Buchanan and Stalder, 2011) and the phylogenetic tree showed that Pietrain and Large White are close, whereas Duroc is far away, thus supporting *F*_*ST*_ results.

Admixture analysis showed similar admixture profiles between G0 and G1. Variations in the proportions of the three pure breed genome are due to random segregation of chromosomes and chromosomal recombinations during the meiosis. After this, MDS plot showed that the G1 population was more dispersed than the G0 population.This results suggests the generation of more diversity in G1 animals than in G0, but, the ROH patterns in G1 animals revealed an increase in autozygosity compared to G0 animals. In G1 animals, ROH have two different origins: either similar breed-specific haplotypes or haplotypes shared between breeds. The ROH patterns observed in G1 animals suggest that random segregation of chromosomes and recombinations during meiosis not only contribute to autozygosity but also to heterozygosity. Indeed, ROH size distribution differs in G1 and pure breeds, we observed fewer large and medium ROH in G1 than in pure breeds due to recombinations. This observation thus confirms the generation of genetic variability at G1.

G1 animals also had more large and medium size ROH than G0 animals. This result shows that some large haplotypes were not homozygous in generation G0 but became homozygous in generation G1 with no breakage due to recombinations. Studying the evolution of these ROH segments in the next generation of the new line would help understand the distribution of recombination events along the genome and would also be interesting with the aim of maximizing diversity in a newly created line.

Our study showed the interest of using ROH to describe diversity in a crossbred population. For the management of diversity, the concept of ROH can be extended to calculate coancestry. de Cara et al. (2013) suggested a method to estimate chromosomal segments shared between two individuals because these segments may be causing ROH in the offspring. So, a mating strategy based on this method limits the generation of ROH in the offspring. Genetic management simulations performed with this method appear to effectively maintain diversity and fitness compared to methods based on marker-by-marker coancestry or genealogical coancestry (de Cara et al., 2013; Bosse et al., 2015). This method could be associated with a monitoring of ROH in the future generations of the new line. Furthermore, when creating a new line, controlling the percentage of allele origin from the founder pure breeds would be a good way to preserve the allele specificity of the different founders. Different methods have been developed to meet this objective, including the breed origin to allele (BOA) approach, which assigns BOA in crossbred animals (Vandenplas et al., 2016).

The next objective of this new line will be the development of a breeding program. But an important question here is when to start selection? Indeed, the crossbred population must be sufficiently mixed and genetically homogeneous before starting the selection. Some authors suggested starting selection after 2 or 3 generations (Legault et al., 1996), but this could be relevant with genomic data to provide information justifying the choice of the starting generation for selection.

### 4.3. Gene Annotation Analysis

In animal breeding populations, selection can influence the fixing and extension of ROH (Kim et al., 2013). The aim of our analysis of gene content in ROH hotspots in G0 animals was to investigate the potential effect of a similar selection that fixed the haplotypes in our three founder breeds and could generate ROH in G0 individuals.

The ROH hotspot on SSC1 carries four interesting genes. First, *IGF1R* (insulin like growth factor 1 receptor) was detected. Pierzchała et al. (2012) showed that the gene expression in the liver of pigs of different breeds was significantly correlated with carcass composition traits, negatively with fat content and positively with meat content. The gene *MEF2A* (myocyte enhancer factor 2A) was identified in a new model of regulation of myogenesis in pigs in which it is hypothesized to play an important role in the balance between intramuscular adipogenesis and myogenesis (Zhao et al., 2011). Then, we detected two genes, *ALDH1A3* (aldehyde dehydrogenase 1 family member A3) and *LRRK1* (leucine-rich repeat kinase 1). When Suwannasing et al. (2018) conducted a GWAS in Large White for different reproduction traits, they found these two genes significantly associated with all studied traits.

On SSC4, we identified an ROH hotspot in a region close to the ROH hotspots identified by Howard et al. (2016) and Szmatoła et al. (2020). In this region, we detected genes *MMP16* (matrix metallopeptidase 16), *CNGB3* (cyclic nucleotide gated channel subunit beta 3), *CPNE3* (Copine 3), *RMDN1* (regulator of microtubule dynamics 1), *WWP1* (WW domain containing E3 ubiquitin protein ligase 1), *SLC7A13* (solute carrier family 7 member 13), and *ATP6V0D2* (ATPase H+ transporting V0 subunit d2) like in the study of Szmatoła et al. (2020). Moreover, this region contains many QTL referenced in PigQTLdb associated with production and meat carcass traits (Hu et al., 2019).

*PLOD2* (procollagen-lysine,2-oxoglutarate 5-dioxygenase 2) on SSC13 codes for a membrane-bound enzyme involved in the formation of extracellular matrix. Four mi-RNAs involved in the inhibition of *PLOD2* are differentially expressed in animals with different muscle development profiles (Ropka-Molik et al., 2018).

On SSC14, in the first ROH hotspot we detected the gene *ALOX5* (arachidonate 5-lipoxygenase). Mehrabian et al. (2008) found this gene to be involved in adiposity-related metabolic pathways. In a second ROH hotspot on SSC14, we identified two genes linked to reproductive traits, *LIF* (LIF interleukin 6 family cytokine) and *GAL3ST1* (galactose-3-O-sulfotransferase 1). *LIF* has two previously studied polymorphisms, one of which had a significant additive effect on number of piglets born alive in German Large White (Spötter et al., 2009). *GAL3ST1* was detected in an ROH hotspot in Large White (Shi et al., 2020) and is hypothesized to be involved in spermatogenesis (Suzuki et al., 2010). In the same genomic region, we also found *INPP5J* (inositol polyphosphate-5-phosphatase J) and *PLA2G3* (phospholipase A2 group III), which are associated with two type of fatty acids (docosahexaenoic acid and n-3 polyunsaturated fatty acid) in Large White (Zappaterra et al., 2018).

The four next genes were detected on the first ROH hotspot on SSC15. *XIRP2* (Xin actin binding repeat containing 2) is involved in the organization of the actin cytoskeleton. In a study comparing transcriptomics data of muscular tissues in Polish Landrace and in Pulawska, a local breed, a mutation in *XIRP2* was detected in Polish Landrace animals but absent in Pulawska animals (Piórkowska et al., 2017). These authors hypothesized that this mutation could cause finer microtubules in Polish Landrace and could be linked to the lesser meat quality observed in the Polish Landrace compared to the local breed. *B3GALT1* (beta-1,3-galactosyltransferase 1) is a membrane-bound glycoprotein. Sun et al. (2016) observed less expression of *B3GALT1* in the liver of animals fed with high fiber diet compared with in the liver of animals fed with a low fiber diet. *STK39* (serine/threonine kinase 39) is an actor of the cellular stress response signaling pathway. In a comparative study between human and porcine species, *STK39* was reported to be significantly associated with subscapular skinfold thickness in human and back-fat thickness in pig (Kim et al., 2012). *CERS6* (ceramide synthase 6) is involved in sphingolipids synthesis. In mice, knock-out of the *CERS6* gene provided protection against obesity (Hammerschmidt et al., 2019). Finally, we detected the gene *NCKAP1* (NCK-associated protein 1) in a second ROH hotspot on SSC15. Hamill et al. (2012) compared transcriptomic profiles of pork meat of varying tenderness and found *NCKAP1* overexpressed in tender meat.

We detected several genes in ROH hotspots in G0 animals. Gene annotation is difficult particularly in large ROH hotspots with a large number of genes, and sometimes no annotation is available. However, we were able to distinguish interesting genomic regions on SSC4, SSC14, or SSC15, which could contain genes under similar selection in the three founder breeds. It could be relevant to characterize with more precision these genes to analyze if some polymorphisms of interest could have been selected.

## 5. Conclusions

The maximization of diversity during the first generations of a new synthetic line is a prerequisite for long-term genetic progress. We have shown that ROH detection is an interesting tool to characterize inbreeding in crossbred animals. ROH persisted in crossbred offspring of a three-way crossbreeding program over two generations. This phenomenon can be explained by haplotype sharing between the three parental breeds. We have observed an increase in genetic diversity between G0 and G1 with an analysis SNP by SNP but we have observed an increase of ROH inbreeding too. This result suggests that it could be interesting to continue the characterization of ROH in next generations of the new line to manage genetic diversity.

## Data Availability Statement

The genotyping data is available at: https://doi.org/10.15454/E6C05S.

## Ethics Statement

Ethical review and approval was not required for the animal study because DNA samples were obtained from breeding company NUCLEUS through its routine practice in the framework of breeding programs. Written informed consent was obtained from the owners for the participation of their animals in this study.

## Author Contributions

AG carried out the analyses and wrote the first version of the manuscript. CL and SR-R supervised the analysis and made major contributions to writing of the article. BL supervised data generation and contributed to writing article. All authors read and approved the final manuscript.

## Conflict of Interest

AG and BL were employed by the company SAS NUCLEUS. The remaining authors declare that the research was conducted in the absence of any commercial or financial relationships that could be construed as a potential conflict of interest.

## References

[B1] Alexander D. Novembre J. Lange K. (2009). Fast model-based estimation of ancestry in unrelated individuals. Genome Res. 19, 1655–1664. 10.1101/gr.094052.109 19648217 PMC2752134

[B2] Bidanel J.-P. (1992). Comment Exploiter la Variabilité génétique entre races: du croisement Simple à la souche synthétique. INRA Productions Animales, 249–254. 10.20870/productions-animales.1992.5.HS.4299

[B3] Biscarini F. Cozzi P. Gaspa G. Marras G. (2019). detectRUNS: Detect Runs of Homozygosity and Runs of Heterozygosity in Diploid Genomes. Guelph: R package version *0.9.6*.

[B4] Bosse M. Megens H.-J. Madsen O. Crooijmans R. P. Ryder O. A. Austerlitz F.. (2015). Using genome-wide measures of coancestry to maintain diversity and fitness in endangered and domestic pig populations. Genome Res. 25, 970–981. 10.1101/gr.187039.114 26063737 PMC4484394

[B5] Bosse M. Megens H.-J. Madsen O. Paudel Y. Frantz L. A. F. Schook L. B.. (2012). Regions of homozygosity in the porcine genome: consequence of demography and the recombination landscape. PLoS Genet. 8:e1003100. 10.1371/journal.pgen.1003100 23209444 PMC3510040

[B6] Buchanan D. S. Stalder K. (2011). Breeds of pigs, in The Genetics of the Pig, eds Rothschild M. F. Ruvinsky A. (CABI), 445–472. 10.1079/9781845937560.0445

[B7] Chang C. C. Chow C. C. Tellier L. C. Vattikuti S. Purcell S. M. Lee J. J. (2015). Second-generation PLINK: rising to the challenge of larger and richer datasets. GigaScience 4:7. 10.1186/s13742-015-0047-8 25722852 PMC4342193

[B8] Cockerham C. C. Weir B. (1984). Covariances of relatives stemming from a population undergoing mixed self and random mating. Biometrics 40, 157–164. 10.2307/2530754 6733226

[B9] Curik I. Ferenčaković M. Sölkner J. (2014). Inbreeding and runs of homozygosity: a possible solution to an old problem. Livestock Sci. 166, 26–34. 10.1016/j.livsci.2014.05.034

[B10] de Cara M. Á. R. Villanueva B. Toro M. Á. Fernández J. (2013). Using genomic tools to maintain diversity and fitness in conservation programmes. Mol. Ecol. 22, 6091–6099. 10.1111/mec.12560 24128280

[B11] Doekes H. P. Veerkamp R. F. Bijma P. de Jong G. Hiemstra S. J. Windig J. J. (2019). Inbreeding depression due to recent and ancient inbreeding in dutch holstein-friesian dairy cattle. Genet. Select. Evol. 51:54. 10.1186/s12711-019-0497-z 31558150 PMC6764141

[B12] Ferenčaković M. Sölkner J. Curik I. (2013). Estimating autozygosity from high-throughput information: effects of snp density and genotyping errors. Genet. Select. Evol. 45:42. 10.1186/1297-9686-45-42 24168655 PMC4176748

[B13] Ganteil A. Cottereau M. Rodriguez-Ramilo S. Ligonesche B. Larzul C. (2020). Diversite genomique de porcs issus d'un croisement large White × Pietrain. Journées de la Recherche Porcine en France. 52, 7–12.

[B14] Gómez Raya L. Rauw W. M. Dunkelerger J. R. Dekkers J. C. M. (2019). Autozygosity and genetic differentiation of Landrace and Large White pigs as revealed by the genetic analyses of crossbreds. Front. Genet. 10:739. 10.3389/fgene.2019.00739 31543894 PMC6739446

[B15] Gorssen W. Meyermans R. Buys N. Janssens S. (2019). SNP genotypes reveal breed substructure, selection signatures and highly inbred regions in Piétrain pigs. Anim. Genet. 51, 32–42. 10.1111/age.12888 31809557 PMC7003864

[B16] Hamill R. M. McBryan J. McGee C. Mullen A. M. Sweeney T. Talbot A.. (2012). Functional analysis of muscle gene expression profiles associated with tenderness and intramuscular fat content in pork. Meat Sci. 92, 440–450. 10.1016/j.meatsci.2012.05.007 22688437

[B17] Hammerschmidt P. Ostkotte D. Nolte H. Gerl M. J. Jais A. Brunner H. L.. (2019). CERS6-derived sphingolipids interact with MFF and promote mitochondrial fragmentation in obesity. Cell 177, 1536–1552. 10.1016/j.cell.2019.05.008 31150623

[B18] Howard J. T. Tiezzi F. Huang Y. Gray K. A. Maltecca C. (2016). Characterization and management of long runs of homozygosity in parental nucleus lines and their associated crossbred progeny. Genet. Select. Evol. 48:91. 10.1186/s12711-016-0269-y 27884108 PMC5123398

[B19] Hu Z.-L. Park C. A. Reecy J. M. (2019). Building a livestock genetic and genomic information knowledgebase through integrative developments of Animal QTLdb and corrDB. Nucleic Acids Res. 47, D701–D710. 10.1093/nar/gky1084 30407520 PMC6323967

[B20] Kim E.-S. Cole J. B. Huson H. Wiggans G. R. Van Tassell C. P. Crooker B. A.. (2013). Effect of artificial selection on runs of homozygosity in US Holstein cattle. PLoS ONE 8:e80813. 10.1371/journal.pone.0080813 24348915 PMC3858116

[B21] Kim J. Lee T. Kim T.-H. Lee K.-T. Kim H. (2012). An integrated approach of comparative genomics and heritability analysis of pig and human on obesity trait: evidence for candidate genes on human chromosome 2. BMC Genomics 13:711. 10.1186/1471-2164-13-711 23253381 PMC3562524

[B22] Legault C. Ménissier F. Ricordeau G. Rouvier R. (1996). Les Lignées Originales de L'inra: Historique, Développement et Impact sur les Productions Animales. INRA Productions Animales, 41–56. 10.20870/productions-animales.1996.9.HS.4085

[B23] Mehrabian M. Schulthess F. Nebohacova M. Castellani L. Zhou Z. Hartiala J.. (2008). Identification of ALOX5 as a gene regulating adiposity and pancreatic function. Diabetologia 51:978. 10.1007/s00125-008-1002-3 18421434 PMC2835627

[B24] Meyermans R. Gorssen W. Buys N. Janssens S. (2020). How to study runs of homozygosity using PLINK? A guide for analyzing medium density SNP data in livestock and pet species. BMC Genomics 21:94. 10.1186/s12864-020-6463-x 31996125 PMC6990544

[B25] Pemberton T. J. Absher D. Feldman M. W. Myers R. M. Rosenberg N. A. Li J. Z. (2012). Genomic patterns of homozygosity in worldwide human populations. Am. J. Hum. Genet. 91, 275–292. 10.1016/j.ajhg.2012.06.014 22883143 PMC3415543

[B26] Peripolli E. Munari D. P. Silva M. V. G. B. Lima A. L. F. Irgang R. Baldi F. (2017). Runs of homozygosity: current knowledge and applications in livestock. Anim. Genet. 48, 255–271. 10.1111/age.12526 27910110

[B27] Pierzchała M. Pareek C. S. Urbański P. Goluch D. Kamyczek M. Różycki M.. (2012). Study of the differential transcription in liver of growth hormone receptor (GHR), insulin-like growth factors (IGF1, IGF2) and insulin-like growth factor receptor (IGF1R) genes at different postnatal developmental ages in pig breeds. Mol. Biol. Rep. 39, 3055–3066. 10.1007/s11033-011-1068-8 21695430

[B28] Piórkowska K. Żukowski K. Szmatoła T. Ropka-Molik K. Tyra M. (2017). Transcript variants of a region on SSC15 rich in QTLs associated with meat quality in pigs. Ann. Anim. Sci 17, 703–715. 10.1515/aoas-2016-0095

[B29] Purfield D. C. McParland S. Wall E. Berry D. P. (2017). The distribution of runs of homozygosity and selection signatures in six commercial meat sheep breeds. PLoS ONE 12:e0176780. 10.1371/journal.pone.0176780 28463982 PMC5413029

[B30] Raudsepp T. Chowdhary B. P. (2011). Cytogenetics and chromosome maps, in The Genetics of the Pig, eds Rothschild M. F. Ruvinsky A. (CABI), 134–178. 10.1079/9781845937560.0134

[B31] Ropka-Molik K. Pawlina-Tyszko K. Żukowski K. Piórkowska K. Żak G. Gurgul A. Derebecka N.. (2018). Examining the genetic background of porcine muscle growth and development based on transcriptome and miRNAome data. Int. J. Mol. Sci. 19:1208. 10.3390/ijms19041208 29659518 PMC5979540

[B32] Schäler J. Krüger B. Thaller G. Hinrichs D. (2020). Comparison of ancestral, partial, and genomic inbreeding in a local pig breed to achieve genetic diversity. Conserv. Genet. Resour. 12, 77–86. 10.1007/s12686-018-1057-5

[B33] Shi L. Wang L. Liu J. Deng T. Yan H. Zhang L.. (2020). Estimation of inbreeding and identification of regions under heavy selection based on runs of homozygosity in a Large White pig population. J. Anim. Sci. Biotechnol. 11:46. 10.1186/s40104-020-00447-0 32355558 PMC7187514

[B34] Spötter A. Müller S. Hamann H. Distl O. (2009). Effect of polymorphisms in the genes for LIF and RBP4 on litter size in two German pig lines. Reproduct. Domestic Anim. 44, 100–105. 10.1111/j.1439-0531.2007.01004.x 18537906

[B35] Sun Y. Yu K. Zhou L. Fang L. Su Y. Zhu W. (2016). Metabolomic and transcriptomic responses induced in the livers of pigs by the long-term intake of resistant starch. J. Anim. Sci. 94, 1083–1094. 10.2527/jas.2015-9715 27065270

[B36] Suwannasing R. Duangjinda M. Boonkum W. Taharnklaew R. Tuangsithtanon K. (2018). The identification of novel regions for reproduction trait in Landrace and Large White pigs using a single step genome-wide association study. Asian Austral. J. Anim. Sci. 31, 1852–1862. 10.5713/ajas.18.0072 29879826 PMC6212738

[B37] Suzuki T. Kosaka-Suzuki N. Pack S. Shin D.-M. Yoon J. Abdullaev Z.. (2010). Expression of a testis-specific form of GAL3ST1 (CST), a gene essential for spermatogenesis, is regulated by the CTCF paralogous gene BORIS. Mol. Cell. Biol. 30, 2473–2484. 10.1128/MCB.01093-09 20231363 PMC2863697

[B38] Szmatoła T. Jasielczuk I. Semik-Gurgul E. Szyndler-Nkedza M. Blicharski T. Szulc K.. (2020). Detection of runs of homozygosity in conserved and commercial pig breeds in Poland. J. Anim. Breed. Genet. 137, 571–580. 10.1111/jbg.12482 32362048

[B39] Tortereau F. Servin B. Frantz L. Megens H.-J. Milan D. Rohrer G.. (2012). A high density recombination map of the pig reveals a correlation between sex-specific recombination and GC content. BMC Genomics 13:586. 10.1186/1471-2164-13-586 23152986 PMC3499283

[B40] Vandenplas J. Calus M. P. Sevillano C. A. Windig J. J. Bastiaansen J. W. (2016). Assigning breed origin to alleles in crossbred animals. Genet. Select. Evol. 48:61 10.1186/s12711-016-0240-y PMC4994281 27549177

[B41] Zanella R. Peixoto J. O. Cardoso F. F. Cardoso L. L. Biegelmeyer P. Cant ao M. E.. (2016). Genetic diversity analysis of two commercial breeds of pigs using genomic and pedigree data. Genet. Select. Evol. 48:24. 10.1186/s12711-016-0203-3 27029213 PMC4812646

[B42] Zappaterra M. Ros-Freixedes R. Estany J. Davoli R. (2018). Association study highlights the influence of ELOVL fatty acid elongase 6 gene region on backfat fatty acid composition in Large White pig breed. Animal 12, 2443–2452. 10.1017/S1751731118000484 29580300

[B43] Zhang J. Song H. Zhang Q. Ding X. (2019). Assessment of relationships between pigs based on pedigree and genomic information. Animal 14, 697–705. 10.1017/S1751731119002404 31708004

[B44] Zhang Q. Calus M. P. Guldbrandtsen B. Lund M. S. Sahana G. (2015). Estimation of inbreeding using pedigree, 50k SNP chip genotypes and full sequence data in three cattle breeds. BMC Genetics 16:88. 10.1186/s12863-015-0227-7 26195126 PMC4509611

[B45] Zhao X. Mo D. Li A. Gong W. Xiao S. Zhang Y.. (2011). Comparative analyses by sequencing of transcriptomes during skeletal muscle development between pig breeds differing in muscle growth rate and fatness. PLoS ONE 6:e19774. 10.1371/journal.pone.0019774 21637832 PMC3102668

